# Comparison of the Effectiveness of Transcranial Direct Current Stimulation (tDCS) and Short-Term Cognitive Rehabilitation Protocols on the Improvement of Depression and Anxiety Symptoms in Patients With Mild Alzheimer's Disease

**DOI:** 10.1155/jare/6616509

**Published:** 2025-07-29

**Authors:** Arezoo Mojarrad, Esmaeil Sadri Damirchi, Ali Sheykholeslami, Ali Rezaeisharif, Vahid Abbasi, Mohammadreza Noroozi Homayoon

**Affiliations:** ^1^Department of Counseling, University of Mohaghegh Ardabili, Ardabil, Iran; ^2^Department of Neurology, Ardabil University of Medical Sciences, Ardabil, Iran

**Keywords:** anxiety, cognitive rehabilitation, depression, mild Alzheimer's, transcranial direct current stimulation

## Abstract

**Aim:** Today, Alzheimer's disease is one of the most common diseases, especially in old age, and it is important to help recognize and treat this disease. The purpose of this study was to compare the effectiveness of transcranial direct current stimulation (tDCS) and short-term cognitive rehabilitation protocols on the improvement of depression and anxiety symptoms in patients with mild Alzheimer's disease.

**Methods:** The research method was an extended experiment with two experimental groups and one control group. The statistical population included all patients over 65 years of age with mild Alzheimer's who had been referred to a neurologist in 2020, and among these people, 60 people were selected through available sampling and then randomly assigned to two experimental groups and one control group. Then, the independent variables of the tDCS method for 10 sessions of 20 min once a week were applied to an experimental group and a short-term cognitive rehabilitation program of 9 sessions (90 min each session) was applied once a week to the second experimental group, and no intervention was performed on the third group. After the end of the intervention, the post-test was conducted with an interval of 1 week on the experimental and control groups. After 1 month, the studied groups were followed up again. A neuropsychological questionnaire (NPI) was used to collect information.

**Results:** The results showed that both studied methods caused a significant reduction in depression in both the post-test and follow-up periods, but only the tDCS method was able to maintain its reduction in the follow-up period. Also, both methods have caused a significant improvement in the anxiety variable both during the post-test and during the follow-up period.

**Conclusion:** Therefore, it can be concluded that both methods can be used to improve the symptoms of depression and anxiety in patients with mild Alzheimer's disease.

## 1. Introduction

The significant increase in life expectancy over recent decades has led to rapid growth in the elderly population worldwide [1]. Aging is an inevitable phenomenon that is part of the natural transformations of life throughout the lifespan [2]. According to the World Health Organization (WHO), individuals aged 60 years and older are considered elderly [3]. According to a United Nations report, life expectancy at age 60 is expected to rise globally from 19.7 years in the period 2045 to 2050. This report predicts that between 2045 and 2050, the population aged over 60 in less developed countries will grow at an annual rate of 5.3%, which is nine times greater than the growth rate of the same population in developed countries. Iran's population has also experienced significant changes in total fertility and mortality rates in recent years, leading to a shift in the age structure from youth to middle age. Continuing this trend will result in a rapid aging of Iran's population [4], posing significant challenges for societies and healthcare systems [5]. As individuals age, they gradually lose some of their physiological, cognitive, and social functions. Although this decline in functional status may not necessarily lead to dependency, it significantly affects the vulnerability of this demographic group [6]. Cognitive impairments are among the common issues of old age, affecting approximately 35% of the elderly, with Alzheimer's disease being considered the progressive stage of this impairment [7]. Alzheimer's disease is characterized by progressive and degenerative brain damage that severely impacts cognitive functions and brain processes, occurring in the context of full consciousness and depending on the type and severity of the underlying cause [8]. Alzheimer's disease manifests as a collection of cognitive, memory, language, psychological, and psychiatric disturbances, disrupting daily activities [9]. Although the progression of Alzheimer's disease varies significantly from patient to patient, there are similarities that allow for classification into mild, moderate, and advanced stages [10]. Research shows that the greater the severity of Alzheimer's disease, the more pronounced the deficiencies in memory and executive functions, which are considered indicators of disease progression [11]. The clinical symptoms of mild Alzheimer's syndrome include noticeable recent memory problems, deficits in at least one other cognitive domain, and reduced independence in functioning. Impairment in functioning can manifest in various forms, such as difficulties in managing finances, spatial disorientation in familiar places, and inability to perform occupational or household tasks. At this stage, patients often have less difficulty recalling past information [10]. Neuropsychiatric symptoms are common among patients with dementia (Alzheimer's disease), with reports indicating a 95% prevalence rate in these patients [12, 13]. Increasing evidence suggests that psychiatric symptoms in Alzheimer's patients are primarily reflective of pathological changes related to the disease [14]. These symptoms, which are fundamental aspects of dementia, have significant clinical implications for the quality of life of both patients and their caregivers [15]. Depression and anxiety are among the most common types of these symptoms, imposing substantial challenges on patients and their caregivers. One of the treatments that can improve cognitive function and neuropsychiatric symptoms is transcranial direct current stimulation (tDCS). The advantages of this treatment include its simplicity, lack of side effects, short duration, low cost, and its nature as a nonpharmacological intervention devoid of the side effects of chemical medications [16]. tDCS is used to control the excitability of neural cells by delivering a small current through an electrode placed on the scalp.

Anodal stimulation increases cortical activity by bringing the resting potential closer to the threshold potential, while cathodal stimulation inhibits excitability by separating the resting potential from the threshold potential [8]. This noninvasive brain stimulation method uses a weak electrical current applied to the scalp to induce temporary changes in the excitability of cortical areas [17]. Applying tDCS to the left dorsolateral prefrontal cortex (DLPFC) increases parasympathetic activity and decreases sympathetic activity, leading to improved cognitive function [18].

Since controlling brain activity impacts brain functions, especially memory, planning, attention, and information processing, researchers are examining the clinical use of the DLPFC for central nervous system disorders and cognitive improvement. Placing the anode on the left DLPFC and the cathode on the right DLPFC is the most common protocol for tDCS to enhance working memory, cognitive performance, and mental state [17]. Studies have shown the promising therapeutic role of noninvasive brain stimulation in neuropsychiatric disorders, including depression and anxiety. For example, Nasiri et al. [19]; Goerigk et al. [20]; and Herrera-Melendez et al. [21] demonstrated the efficacy of this treatment in improving depressive and anxiety symptoms in various patients. Another type of intervention that has recently attracted the attention of many researchers and therapists is cognitive intervention, known as cognitive rehabilitation. Cognitive rehabilitation is a term used for the treatment and rehabilitation of cognitive disorders, with the primary goal of improving deficits and cognitive functions such as memory, executive functions, social cognition, and attention [22]. Cognitive rehabilitation is based on principles of neuroplasticity and includes targeted exercises to improve various cognitive domains such as memory, attention, language, and executive functions. It involves training based on findings from cognitive sciences to enhance or restore cognitive functions (accuracy, attention, visual-spatial perception, auditory discrimination, various types of memory, especially working memory, and other executive functions). This approach relies on the principle of neuroplasticity and aims to restore lost cognitive capacities through targeted exercises and stimuli, ultimately enhancing individual performance in activities through improved perception, attention, memory, problem-solving, alertness, and conceptualization [23]. Studies such as those by Sayadi et al. [24], Jafari and Bafandeh [25], and Olukolade and Osinowo [26] have shown the effectiveness of these cognitive programs and training in improving psychological symptoms like depression and anxiety. Considering that caring for Alzheimer's patients requires significant time and financial resources, causing various problems for the patient and their family, early diagnosis and treatment of this disease can slow the progression, delay functional decline, reduce treatment costs, and alleviate the burden on caregivers [27]. Additionally, there have been limited studies on the effectiveness of tDCS on the prefrontal cortex in elderly Alzheimer's patients in Iran, and most cognitive rehabilitation protocols are long-term and require numerous sessions. This study aims to address whether there is a significant difference between the effectiveness of cognitive rehabilitation programs and tDCS on depressive and anxiety symptoms in patients with mild Alzheimer's disease.

## 2. Materials and Methods

This research utilizes an expanded experimental method with two experimental groups and one control group, conducted using a pre-test and post-test approach. The statistical population of this study includes all elderly individuals over the age of 65 with mild Alzheimer's disease who visited a neurologist in the year 2022. The randomization was carried out using a computer-based simple randomization method via the software Randomizer.org. After confirming eligibility based on the inclusion criteria, 60 qualified participants were randomly assigned equally (20 participants per group) using a table of random numbers, ensuring a balanced distribution of baseline characteristics such as age and severity of Alzheimer's symptoms. Due to the nature of the interventions, complete blinding of participants and therapists was not feasible, as the differences between the tDCS sessions, cognitive rehabilitation program, and the absence of intervention in the control group were evident. However, the outcome assessors, who administered the Neuropsychiatric Inventory (NPI) during the pre-test, post-test, and follow-up phases, were blinded to the group allocation of participants (single-blind design) in order to minimize assessment bias. The independent variable, tDCS, was administered to one experimental group for 10 sessions of 20 min each, once a week. tDCS was delivered using a two-channel NEUROSTIM 2 device (Medina Teb Gostar, Iran), equipped with two independent power sources to provide fully isolated anodal and cathodal stimulation. The protocol involved anodal stimulation applied to the left DLPFC (DLPFC, F3 position per the 10–20) and cathodal stimulation to the right DLPFC (F4 position) to enhance cortical excitability and modulate emotional processing. Two conductive carbon electrodes (5 × 7 cm), encased in saline-soaked (0.9% NaCl) synthetic sponges, were used to ensure optimal conductivity and minimize skin irritation. A constant current of 2 mA was delivered for 20 min per session, with a 30 s linear ramp-up phase at the start and a 30 s linear ramp-down phase at the end to enhance participant comfort and safety. The device's impedance control feature maintained impedance below 10 kΩ, preventing skin irritation or burns. The intervention consisted of 10 weekly sessions, each lasting approximately 25 min (including setup), administered by a trained clinician following standardized safety protocols. The second experimental group received a short-term cognitive rehabilitation program consisting of 9 sessions (each session lasting 90 min), designed based on Luria's approach to the substitution of healthy functions [28], as outlined in Table 1. The content validity of the program was confirmed by experts, and it was administered once a week. No intervention was performed on the control group. After the intervention, a post-test was conducted on both experimental and control groups 1 week later. Additionally, the groups were followed up 1 month after the intervention. Inclusion criteria for the study were: absence of heart disease, no wounds or scratches on the head, informed consent to participate in the study, absence of contagious diseases, absence of serious illnesses such as cancer, and absence of epilepsy. Exclusion criteria included the presence of personality disorders, a history of substance abuse or dependency on drugs or alcohol, and receiving psychological services from other centers during the study. To control for potential confounding factors, participants' medication profiles, including antidepressants, anxiolytics, and Alzheimer's-related medications (e.g., cholinesterase inhibitors), were documented at baseline through caregiver interviews and medical record reviews. Participants were required to maintain stable medication regimens throughout the study, and any medically necessary changes were recorded and considered in the analysis. Cognitive reserve was approximated using participants' educational attainment, a recognized proxy for cognitive reserve, collected via structured interviews. Participants who initiated new psychotropic medications or experienced significant dosage changes during the study were excluded from the final analysis to minimize medication-related confounding effects. It is worth noting that ethical considerations were observed by explaining the purpose of the study and the principle of confidentiality to the participants and obtaining their informed consent before enrolling them in the study. The following questionnaire was used to collect data.

### 2.1. NPI Questionnaire

The NPI questionnaire is used to assess nonpsychological symptoms in dementia patients and is also applicable to vascular dementia and other neurological diseases in the elderly. The designers of this questionnaire have added a caregiver distress scale to it in separate studies. The NPI includes 12 subscales, with scores higher than 6 indicating the presence of the respective subscale in the participant [29]. Compared to other tools for assessing psychological symptoms, the NPI offers several useful features, such as covering a broader range of psychopathological symptoms, evaluating common behavioral changes associated with dementia (including irritability, euphoria, and apathy, in addition to other behavioral changes assessed by other tools), and providing screening questions. Its depression and anxiety subscales are particularly suited for this study, as they sensitively capture symptom severity and frequency in Alzheimer's disease, enabling precise evaluation of intervention-related changes based on caregiver reports. One significant advantage is that scoring the 12 NPI items is based on information obtained from caregivers, thus avoiding the issues related to asking questions directly to the patient or relying on observations of the patient's behaviors over a short period. The score for each disorder is obtained by multiplying the frequency (rated 1–4) and severity (rated 1–3) scores of the disorder in the patient. The total score for psychological symptoms in a patient is determined by summing the scores of the identified disorders. High scores indicate a high frequency and severity of psychological symptoms in the patient [30]. These tools have demonstrated excellent content and construct validity alongside acceptable reliability. The test-retest reliability correlations for symptom frequency and severity were reported as 0.79 and 0.86, respectively [12]. Additionally, inter-rater reliability showed Pearson correlation coefficients of 0.987 for aggression, 0.953 for depression, 0.975 for anxiety, 1.00 for apathy, 0.997 for disinhibition, 0.944 for irritability, 1.00 for appropriate motor behavior, 1.00 for sleep pattern changes, 0.991 for appetite and eating pattern changes, and 0.968 for the overall questionnaire score (*p* < 0.005). The internal consistency, measured by Cronbach's alpha, was 0.639 for all 12 subscales and the overall questionnaire score, and 0.601 for the first 10 subscales and the overall questionnaire score. In test-retest reliability, Pearson correlation coefficients for various subscales were as follows: 1.00 for delusions, 1.00 for hallucinations, 0.984 for aggression, 0.973 for depression, 0.939 for anxiety, 1.00 for euphoria, 0.915 for apathy, 1.00 for disinhibition, 0.950 for irritability, 1.00 for abnormal motor behavior, 0.936 for sleep, 1.00 for appetite, and 0.961 for the overall questionnaire score (P for irritability was 0.131, and for other variables less than 0.005) [29].

## 3. Findings

Descriptive information (mean and standard deviation) related to the variables of depression and anxiety, categorized by the control group, short-term cognitive rehabilitation program group, and the tDCS group at three different times (pre-test, post-test, and follow-up period), is reported in Table 2.

To assess the effectiveness of the two studied methods in improving depression and anxiety, a repeated measures analysis of variance (ANOVA) was conducted with three groups (control, short-term cognitive rehabilitation program, and transcranial direct current stimulation (tDCS)) and three different times (pre-test, post-test, and follow-up period). It should be noted that all assumptions for this test were met, and the normality of the residuals for all models was tested using the Shapiro–Wilk test, which was not rejected at a 0.95 confidence level. In this analysis, Mauchly's test of sphericity was initially used to test the null hypothesis of sphericity. If the significance of this test was greater than 0.05, the null hypothesis of sphericity was accepted, and the ANOVA was conducted assuming sphericity. Otherwise, if sphericity was not assumed, depending on whether the epsilon value was greater than 0.75 or not, the Huynh–Feldt correction or the Greenhouse–Geisser correction was applied, respectively. The results of the Mauchly's test and the test of equality of means for the variables of depression and anxiety are reported in Table 3.

The results in Table 3 indicate that there is a significant difference in the two variables, depression and anxiety, across the three times (pre-test, post-test, and follow-up). The effect sizes (eta-squared) for the variables of depression and anxiety were 0.805 and 0.809, respectively. Additionally, to compare the means between the three times and separately for the three groups, the Bonferroni post hoc test was used, and the results are reported in Table 4.

The results reported in Table 4 indicate that both the short-term cognitive rehabilitation method and the tDCS method significantly reduced depression in both the post-test and follow-up periods. The only difference between the two methods regarding this variable is that, unlike the tDCS method, the short-term cognitive rehabilitation method did not maintain its reduction effect in the follow-up period. Furthermore, both methods significantly improved anxiety in both the post-test and follow-up periods.

## 4. Discussion and Conclusion

This study aimed to compare the effectiveness of tDCS and a short-term cognitive rehabilitation protocol on depression and anxiety in patients with mild Alzheimer's disease. The first finding showed that both methods significantly reduced depression in both the post-test and follow-up periods. In simpler terms, both methods effectively reduced depression in patients with Alzheimer's disease. The only difference was that the short-term cognitive rehabilitation method failed to maintain its reduction effect in the follow-up period, unlike the tDCS method. This finding aligns with the results of studies by Nasiri et al. [19]; Goerigk et al. [20]; Herrera-Melendez et al. [21]; Nozari et al. [31]; and Berryhill and Martin [32].

Another finding showed that both the short-term cognitive rehabilitation method and tDCS significantly improved anxiety in both the post-test and follow-up periods. In simpler terms, both methods equally reduced anxiety in patients with mild Alzheimer's disease, with no significant difference in their effectiveness. This finding is consistent with the studies by Bashi Abdolabadi et al. [33]; Sayadi et al. [24]; Jafari and Bafandeh [25]; Olukolade and Osinowo [26]; Taherifard et al. [34]; Arastoo et al. [35]; and Brunoni et al. [36].

To explain the effectiveness of tDCS in reducing depression, it can be stated that recent neuroimaging and electrophysiological studies provide direct evidence for the mechanisms underlying the therapeutic effects of tDCS and cognitive rehabilitation in mild Alzheimer's disease. For instance, anodal tDCS to the left DLPFC enhances cortical excitability and functional connectivity in frontoparietal networks, as evidenced by fMRI, which may improve emotional regulation and reduce depressive symptoms. Additionally, tDCS upregulates brain-derived neurotrophic factor (BDNF), promoting synaptic plasticity and supporting mood improvements in neurodegenerative conditions [37]. Studies have also indicated that right frontal alpha band asymmetry is inversely related to depression [38]. Regarding the effectiveness of tDCS in reducing anxiety, it can be explained that tDCS, by affecting amygdala activity, promotes positive emotional processing and emotional control [39, 40]. The system works such that when the stimulating electrode is placed on the left hemisphere and the inhibitory electrode on the right hemisphere, it reduces negative emotions. This method leads to changes in brain function by altering neuronal excitability [41]. Although the observed symptom improvements in patients may be attributed to interventions such as tDCS, the multifactorial etiology of cognitive decline in Alzheimer's disease—including neurodegeneration, vascular alterations, and psychological factors—suggests that attributing these improvements solely to such interventions oversimplifies the underlying mechanisms. It is more likely that these treatments alleviate depression and anxiety symptoms by modulating neural networks, such as the DLPFC, rather than by directly altering the core neuropathology of Alzheimer's disease (e.g., amyloid-beta or tau accumulation). Psychological factors, such as reduced stress and improved mood, may also indirectly contribute to enhanced quality of life, thereby influencing the outcomes observed. Future studies should explore the interaction between these interventions and biological markers (e.g., CSF amyloid-beta) to clarify their impact on disease pathology [42]. Additionally, the effectiveness of the short-term cognitive rehabilitation method can be explained by its significant impact on improving processing speed, cognitive flexibility, and memory. This method notably enhances the activity of the prefrontal cortex. It is a type of brain training that uses a specific and specialized program aimed at increasing cognitive skills or developing cognitive abilities through exercises that can lead to significant changes in behavioral levels, attention, memory, and other executive functions of the brain. Given the close relationship between executive functions and anxiety disorders, cognitive rehabilitation training can effectively improve anxiety symptoms and reduce anxiety [43]. Regarding the effectiveness of this method in reducing depression, it can be stated that individuals with depression often exhibit frontal lobe dysfunction, which is responsible for the brain's executive functions. It is not surprising that these individuals experience reduced attention, concentration, working memory, and slower information processing, leading to lower overall cognitive performance. Improving these cognitive functions through cognitive rehabilitation training, in addition to enhancing attention, concentration, and information processing, also reduces depression [44], hallucinations and delusions [45]. One of the limitations of this study is the use of convenience sampling. Therefore, it is recommended that future research employ random sampling methods to increase accuracy. Additionally, it is suggested that the effectiveness of a combined method of tDCS and short-term cognitive rehabilitation be tested. Another limitation was the partial control of confounders, such as medication effects and cognitive reserve. Although medication profiles were monitored, individual pharmacological variability may have influenced results. Similarly, cognitive reserve, approximated by educational attainment, may not fully capture this construct. Future studies should use tools like the Cognitive Reserve Index questionnaire (CRIq) and advanced statistical methods, such as analysis of covariance, to better control these factors. Additionally, the difference in session duration and number between tDCS (10 sessions, 20 min each) and cognitive rehabilitation (9 sessions, 90 min each) may introduce a confounding variable, potentially affecting the comparability of intervention effects. Future studies should standardize session duration. Furthermore, this study lacked objective biomarkers, such as amyloid-beta or tau levels, limiting insights into biological mechanisms. Complete blinding was not feasible due to intervention differences, and the 1-month follow-up may not capture long-term effects. Future studies should incorporate biomarkers (e.g., CSF amyloid-beta, tau, or fMRI connectivity), employ sham-controlled tDCS for robust blinding, control confounders using advanced statistical methods, and extend follow-up periods (e.g., 6–12 months) to assess the durability and mechanistic basis of therapeutic effects.

## Figures and Tables

**Table 1:** Cognitive skills enhancement-based rehabilitation protocol.

Sessions	Objective	Content	Duration (min)
1	Introduction to the patient, familiarizing the patient with the treatment environment and therapist, establishing rapport, conducting a pre-test	Mutual introduction of the patient and therapist, familiarizing the patient with the treatment environment, discussing the treatment process, preparing the patient, establishing rapport, conducting a pre-test	90
2	Memory enhancement	Remembering images, recalling names of individuals, chunking	90
3	Memory enhancement exercise, improving attention and orientation skills	Remembering events, performing the “count the ‘and's” exercise, hidden words (part 1)	90
4	Attention and orientation skills exercise, enhancing verbal fluency skills	Hidden words (part 2), letter cues exercise, category cues exercise	90
5	Verbal fluency skills exercise, enhancing language and thinking skills	Animals and colors category exercise, reversing words, proverbs, category differentiation (part 1)	90
6	Language and thinking skills exercise, enhancing visuospatial skills	Category differentiation (part 2), logo matching, remembering patterns, matching traffic signs	90
7	Review of memory, attention, and orientation skills	Iranian restaurant exercise, ordering and shopping from the supermarket, finding Persian numbers among words	90
8	Review of verbal fluency, language, and visuospatial skills	Name cues exercise, proverbs and situations, animal matching	90
9	Closure and conducting a post-test	Final discussion with the patient and conducting the closing ceremony, post-test	90

**Table 2:** Descriptive statistics of depression and anxiety variables by experimental group and time; (standard deviation) mean.

Variable	Mauchly's test		Equality of means test		
	Significance	Epsilon	Test used	Significance (effect size)	
				Time	Group ∗ time
Depression	0.001	0.640	Greenhouse–Geisser	(0.805) 0.001	(0.740) 0.001
Anxiety	0.001	0.769	Huynh-Feldt	(0.809) 0.001	(0.760) 0.001

**Table 3:** Results of Mauchly's test and test of equality of means.

Variable	Mauchly's test		Equality of means test		
	Significance	Epsilon	Test used	Significance (effect size)	
				Time	Group ∗ time
Depression	0.001	0.640	Greenhouse–Geisser	(0.805) 0.001	(0.740) 0.001
Anxiety	0.001	0.769	Huynh–Feldt	(0.809) 0.001	(0.760) 0.001

**Table 4:** Bonferroni post-hoc tests between three times for each study group.

Variable	Bonferroni post hoc test results (pairwise comparison of means)		
	Control group	Short-term cognitive rehabilitation	TDCS
Depression	—	Pre-test-post-test (*p*=0.001) Pre-test-follow-up (*p*=0.001) Post-test-follow-up (*p*=0.008)	Pre-test-post-test (*p*=0.001) Pre-test-follow-up (*p*=0.001)

Anxiety	—	Pre-test-post-test (*p*=0.001) Pre-test-follow-up (*p*=0.001)	Pre-test-post-test (*p*=0.001) Pre-test-follow-up (*p*=0.001)

## Data Availability

The datasets generated and/or analyzed during the current study are available from the corresponding author on reasonable request.
